# MAIT Cells and Microbiota in Multiple Sclerosis and Other Autoimmune Diseases

**DOI:** 10.3390/microorganisms9061132

**Published:** 2021-05-24

**Authors:** Rosella Mechelli, Silvia Romano, Carmela Romano, Emanuele Morena, Maria Chiara Buscarinu, Rachele Bigi, Gianmarco Bellucci, Roberta Reniè, Giulia Pellicciari, Marco Salvetti, Giovanni Ristori

**Affiliations:** 1 Department of Human Science and Promotion of Quality of Life, San Raffaele Roma Open University, 00166 Rome, Italy; rosella.mechelli@uniroma5.it; 2 IRCCS San Raffaele Pisana, 00166 Rome, Italy; 3 Centre for Experimental Neurological Therapies (CENTERS), Department of Neurosciences, Mental Health and Sensory Organs, Sapienza University of Rome, 00189 Rome, Italy; silvia.romano@uniroma1.it (S.R.); carmela.romano8@gmail.com (C.R.); mor.emanuele@gmail.com (E.M.); mchiara.buscarinu@gmail.com (M.C.B.); rachele.bigi@uniroma1.it (R.B.); bellucci.1638116@studenti.uniroma1.it (G.B.); roberta.renie@gmail.com (R.R.); giuliapellicciari11@gmail.com (G.P.); giovanni.ristori@uniroma1.it (G.R.); 4 IRCCS Istituto Neurologico Mediterraneo (INM) Neuromed, 86077 Pozzilli, Italy; 5 Neuroimmunology Unit: IRCCS Fondazione Santa Lucia, 00179 Rome, Italy

**Keywords:** autoimmune disease, mucosal-associated invariant T cells, microbiota, multiple sclerosis, systemic lupus erythematosus, inflammatory arthritis, inflammatory bowel diseases, type 1 diabetes

## Abstract

The functions of mucosal-associated invariant T (MAIT) cells in homeostatic conditions include the interaction with the microbiota and its products, the protection of body barriers, and the mounting of a tissue-repair response to injuries or infections. Dysfunction of MAIT cells and dysbiosis occur in common chronic diseases of inflammatory, metabolic, and tumor nature. This review is aimed at analyzing the changes of MAIT cells, as well as of the microbiota, in multiple sclerosis and other autoimmune disorders. Common features of dysbiosis in these conditions are the reduced richness of microbial species and the unbalance between pro-inflammatory and immune regulatory components of the gut microbiota. The literature concerning MAIT cells in these disorders is rather complex, and sometimes not consistent. In multiple sclerosis and other autoimmune conditions, several studies have been done, or are in progress, to find correlations between intestinal permeability, dysbiosis, MAIT cell responses, and clinical biomarkers in treated and treatment-naïve patients. The final aims are to explain what activates MAIT cells in diseases not primarily infective, which interactions with the microbiota are potentially pathogenic, and their dynamics related to disease course and disease-modifying treatments.

## 1. Introduction

Mucosal-associated invariant T (MAIT) cells represent an important subset of innate-like immune effectors, prevalently present in humans in blood, mucosal surfaces, and the liver [1]. They were initially identified for their invariant T cell receptor-a (TCR-a) chain (Va7.2-Ja33) in T cells from human blood [2]. Then the main features of MAIT cells were defined by consistent investigations: recognition of vitamin B2 (riboflavin) precursor derivatives in the context of MHC class I related molecule (MR1) [3,4]; response to derivatives of microbial origin (bacteria and yeast), and in particular MAIT cell dependency on commensal microbiota, evidenced by their absence from the periphery of germ-free mice [3]; immediate innate-like effector activities in various tissues given their capability of responding to activating cytokines such as interleukin (IL)12, IL23, IL18, and their propensity to execute essential functions in human mucosal and skin barriers (such as secretion of proinflammatory cytokines, cytotoxicity, production of chemokines, and tissue repair [5]).

Vitamin B2 metabolites produced by the microbiota control the thymic development of MAIT cells [6]. Then MAIT cells travel through body fluids toward peripheral tissues, including lymphoid organs, liver, skin, and mucosal barriers. Their functions in homeostatic condition are therefore sensing the microbiota and its products, providing barrier protection, mounting a tissue-repair response in return to injuries or infections. The function seems non-redundant since a T cell subset expressing a homologous TCRα chain was described in mice and cattle, indicating evolutionary conservation of this important role [7].

Dysfunction of blood MAIT cells and dysbiosis are common in chronic diseases of an inflammatory, metabolic, and tumor nature [8]. This review is aimed at analyzing the relationship between the MAIT cells and the microbiota, as well as the changes of MAIT cells in multiple sclerosis (MS) and other autoimmune disorders, such as type 1 diabetes (T1D), inflammatory bowel disease (IBD), rheumatoid arthritis (RA) and systemic lupus erythematosus (SLE) and Sjögren syndrome (SS). Though the literature is rather complex, and sometimes not consistent, it seems useful to point out the main results on the exact role(s) played by MAIT cells in the above conditions. This will pave the way for translational approaches, especially considering the recent great progress on the topic of the interaction between the mucosal immune response and the microbiota in chronic inflammatory diseases.

## 2. The Role of the Microbiota in Shaping MAIT Cells

The development of MAIT cells occurs during a specific temporal window, being impaired outside this developmental period: exposure to microbes that synthesize riboflavin-derived antigens promotes MAIT cell development within the first few weeks of life. In particular, commensal bacteria induce MAIT intra-thymic development, as MAIT cells do not recirculate to the thymus. The MAIT antigen 5-(2-oxopropylideneamino)-6-d-ribitylaminouracil (5-OP-RU), produced by the microbiota, travels from mucosal surfaces to the thymus, where it is captured by MR1 and presented to this innate T subset that eventually becomes mucosally targeted. In adults MAIT cells of body surfaces produce IL-17A and can respond to commensals in an IL-1^-^, IL-18^-^, and antigen-dependent manner, mediating wound healing and responses to injury [9].

Microbiota-derived signals affect MAIT cell biology: besides intra-thymic selection, subsequent expansion and functions in tissues depend on microbiota composition and host conditions. MAIT cells can integrate multiple signals and act through several immune effector functions, in particular related to defense against infectious pathogens and the improvement of wound healing in the body surfaces. A model emerges whereby MAIT cells protect epithelium integrity, being capable of distinguishing between different signals, attacking armful microbes, or favoring homeostasis in the presence of local commensals [6].

Blood MAIT cell frequency is modified during several auto-immune diseases, which are often associated with microbiota dysbiosis (see below), emphasizing the interaction between MAIT cells and the microbiota [10]. In fact, MAIT cells can perceive microbial stress due to changes in riboflavin utilization in various microbial communities. On the other hand, riboflavin metabolites’ availability plays a central role for the MAIT cell-activating potential of diverse microbiota: low MAIT cell-activating potential was associated with high microbial diversity and a high level of riboflavin demand and vice versa [11]. MAIT cells can also discriminate and categorize complex human microbiota through computation of TCR signals depending on antigen load and presenting cells: a T-T cell presentation has been reported, being conventional T cell capable of expressing MR1; this antigen presentation elicits MAIT cell response other than those stimulated by other professional antigen-presenting cells. These functional modulations serve fine-tuned responses to different conditions, including the different activating properties of the microbiota from different body surfaces, or from diverse micro-environmental states of the small intestine compared to those of the colon [11].

## 3. Microbiota Alterations in MS and Autoimmune/Inflammatory Diseases

**Figure 1:**
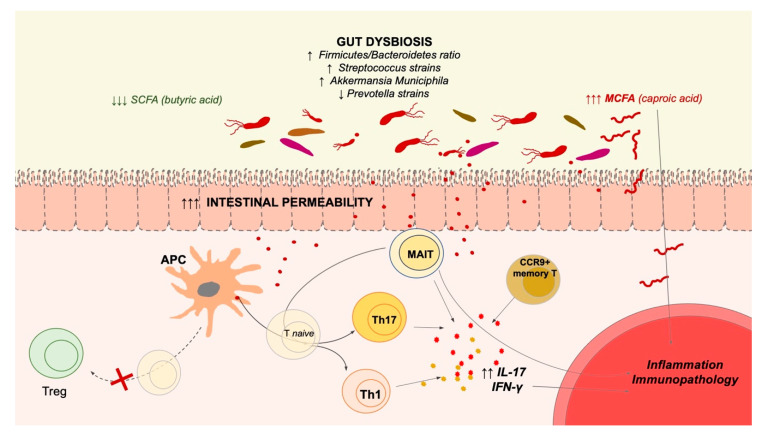
Gut dysbiosis in multiple sclerosis. Abbreviations: SCFA=short chain fatty acid; MCFA=medium chain fatty acid; Treg= T regulatory cell; APC=antigen presenting cell; Th=T helper cell; IL-17=interleukin 17; IFN-γ= interferon gamma. Upward or downward arrows denote respectively increase or decrease of figure components.

The above relationships between MAIT cells and microbiota fit in the interaction between intestinal permeability changes, dysbiosis, and autoimmunity, which has recently been recognized as an important contributor to MS and other immune-mediated disorders [12]. After several studies on animal models of MS, that showed the importance of the gut for the development of neuroinflammation, the role of microbiota changes have recently been evaluated in human disease (Figure 1). Microbiota changes (in particular, the decrease of *Prevotella* strains in the small intestine) have been associated with disease activity and increase of pro-inflammatory T helper 17 immune responses [13], or to alterations in the gut-homing CCR9^+^ memory T cells that correlated with the development of the progressive phase of MS [14]. Other authors reported impaired immunomodulatory properties of the spore-forming fraction from MS patients compared to controls [15]. Along this line, the drop of butyric acid producers (that have anti-inflammatory and gut-barrier enhancer properties) has been described in Caucasoid and Chinese patients [16,17]. Recent works showed specific microbiota changes according to disease phases or phenotypic subtypes of MS [18,19], paving the way to possible tailored approaches to restore an anti-inflammatory microbiota. Recent trials with probiotics or a short fatty chain acid showed possible synergistic effects with current MS therapy [20,21], while material coming from feces of patients was tested in mouse models of disease and proved to be capable of precipitating [22] or improving [23] neuroinflammation. Moreover, *Akkermansia* strains coming from MS patients can ameliorate disease in EAE suggesting a compensatory beneficial response in the microbiome during disease [24].

Studies performed on both human subjects and animal models showed that alteration of gut microbiota composition is related also to other autoimmune diseases such as SLE [25,26,27]), RA [28], [29], SS [30], T1D, and IBD [31]. Common features are the reduced richness of microbial species and a reduced ratio of *Firmicutes/Bacteroides* in the gut.

Mouse model prone to develop SLE showed a reduced community richness with respect to the control mice and a significant change in the distribution of immune cells and expression of some lupus susceptibility genes [32]. In patients with SLE, a lower ratio of *Firmicutes/Bacteroides* was observed and this dysbiosis is associated with higher levels of dsDNA and histone antibodies and with a local inflammatory response [33,34]. Among the *Firmicutes*, a reduction of *Lactobacillaceae* abundance and an increase of *Lachnospiraceae* were associated with the disease condition. Supplementation with *Lactobacillus delbrueckii* and *Lactobacillus rhamnosus* tested in SLE-induced mice, showed the enhancement of regulatory T cells, the decrease of inflammatory cytokines, and the decrease of disease severity [35]. Moreover, dendritic cells obtained from SLE patients and treated *ex-vivo* with probiotics reduced the expression of costimulatory molecules and other surface markers, displaying a tolerogenic phenotype [36]. Other evidence, demonstrated a beneficial effect of probiotics in female mice with lupus nephritis, inducing an anti-inflammatory phenotype and an improvement of intestinal permeability, suggesting a role for sex hormones on the regulatory function of microbiota in lupus [26].

The SS, another diffused rheumatoid disease, share with SLE a reduced richness and composition of gut microbiota. van der Meulen and colleagues observed a significant increase of *Bacteroides vulgatus*, *Bacteroides uniformis*, and *Bacteroides ovatus* in both primary SS and SLE patients with respect to the general population [37]. These microbial alterations did not correlate with the serum level of disease-associated autoantibodies [37,38]. Analyzing the microbiota of the oral cavity, significant differences emerge: a higher level of richness and diversity were observed in healthy controls and SLE patients with respect to the SS [37,39,40], suggesting that the oral dryness can shape the oral microbiome [41,42,43]. Some attempts have been made to evaluate the possible use of probiotics and fecal transplant as therapeutic approaches in SS, without obtaining convincing results [44,45].

In RA patients the decreased gut microbial diversity seems to be correlated with the disease duration and the autoantibodies levels [28,29], two important parameters that should be considered for patient management. In the microbiome of early RA patients was observed a significant increase of *Prevotella* genus [46] and *Prevotella copri* species [47,48], as well as a proliferation of *Bacillus* and *Lactobacillus* [49] in comparison to healthy controls. Moreover, *Prevotella copri* isolated from RA patients can worsen inflammation in mice, which could support a causal role [47]. Treatment with probiotics did not reach conclusive results on their possible beneficial effect [50]: *Lactobacillus casei* and *Lactobacillus acidophilus* have shown to decrease inflammatory markers and disease activity [51,52], while *Lactobacillus rhamnosus* and *Lactobacillus reuteri* did not have significant effects on clinical improvement in active RA patients [53,54].

Analysis of gut microbiota in children before the onset of T1D, highlight a dysbiosis condition [55]. Several studies, performed on children at different time points with respect to the disease onset, have revealed differences in the composition of microbial population within these two predominant phyla in the gut microbiome (*Firmicutes* and *Bacteroidetes*) when compared to healthy controls [56,57]. Moreover, evidence obtained from animal studies have suggested a causal link between intestine microflora and T1D development. These studies proved the efficacy of probiotic supplementation [58], antibiotic use [59], fecal transplant treatment [60], and diet intervention [61] in modifying the risk of T1D by changing the gut colonization patterns. However, translating these findings into achievable therapeutic approaches might be challenging in human disease due to the difficulties in controlling confounding factors.

Many studies, both clinical and experimental, showed that dysbiosis may play a key role in the pathogenesis of IBD [62]: specifically, a reduction of bacteria with anti-inflammatory properties and an increased level of bacteria with pro-inflammatory effects were observed. For instance, *Faecalibacterium prausnitzii* (belonging to the *Clostridium* cluster IV), known for its anti-inflammatory functions by producing butyrate [63], is reduced in the gut of persons with Crohn’s disease (CD) and is associated with a remission condition in Ulcerative colitis (UC) [64]. Among the pro-inflammatory bacteria, a relative increase in Proteobacteria was observed in fecal samples of CD patients, mainly adhesion-invasive *E. coli* [65]. The ability to adhere to the intestinal epithelium affects the permeability and the composition of the gut microbiota, inducing an inflammatory response leading to intestinal inflammation [66]. In the IBDs the studies aimed at evaluating the possible therapeutic effect of probiotics and fecal transplant did not obtain consistent results [62].

## 4. MAIT Cells in MS and Other Autoimmune Diseases

The first work suggesting a pathogenic role of T cell subsets encompassing the MAIT cell subtype in MS was published at the beginning of the last decade, starting from an experimental setting aimed at analyzing blood transcriptomes in disease-discordant monozygotic twins. By combining several approaches, the authors showed that patients with MS presented an expansion of proinflammatory CD161^high^CD8^+^ T cells in the peripheral blood and that CD16^+^CD8^+^ T cells were detectable in the brain immune infiltrates [67]. These data were in agreement with contemporary works showing a lack of differences between MS-discordant monozygotic twins in peripheral blood CD4^+^ T cells, and the increasing appreciation of the role of CD8^+^ T cells as pathogenic effectors in MS [68]. The fact that the International Multiple Sclerosis Genetics Consortium had reported an association between CD161 genetic variants and the disease [69] and that differences in CD161 expression were detected in MS-discordant monozygotic twins, suggested that this alteration affected MS risk through a complex interaction between heritable and non-heritable factors. The CD161^+^CD8^+^ T cells prevalently included chemokine (C-C motif) receptor 6 (CCR6)-positive, cytokine-producing, effector-memory T cells with proinflammatory profiles. The subset included virtually all circulating IL17^+^CD8^+^ T cells, whose proliferation and interferon-gamma production were facilitated by IL12. CCR6^+^ Th17 cells were known to be required for the initiation of the animal model of MS, an effect largely due to the control by CCCR6 of the immune surveillance in the CNS [70]. In fact, CD161^+^CD8^+^CD3^+^ T cells producing interferon-gamma were part of intralesional immune infiltrates and ectopic B cell follicles in post-mortem MS brains.

In the same year as the above work, another group published a study suggesting a potential regulatory role of MAIT cells in neuroinflammation, through suppression of pathogenic Th1 cells [71]. Previous works from the same group had reported results showing a disease-suppressive role of MAIT cells in experimental autoimmune encephalomyelitis (EAE; [72]). In 2016, a work by Salou et al. questioned the pathogenic role of MAIT cells in MS, suggesting that this subset might represent a minor component of the inflammatory process. The MAIT cell subset resulted to represent a low percentage of the total infiltrating T cells in CNS lesions, though the high over-expression of MR1 molecules (that present cognate antigen to MAIT cells), as well as of the activating cytokines IL18 and IL23 suggested that the MS brain represented a suitable microenvironment for MAIT cells pathogenic actions [73].

**Figure 2:**
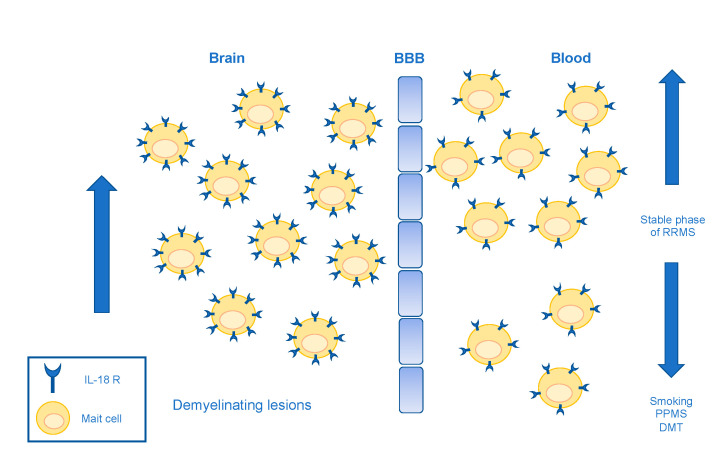
MAIT cell dynamics in multiple sclerosis. Abbreviations: MAIT = mucosal-associated invariant T cells; RRMS = relapsing-remitting multiple sclerosis; PPMS=primary progressive multiple sclerosis; DMT = disease-modifying therapies; IL18 R = interleukin 18 receptor; BBB = blood-brain barrier.

Despite some contradictory reports, a large body of recent evidence confirmed the involvement of MAIT cells in MS pathogenesis. A work confirming the dependence of this T cell subset on IL18 may help, at least in part, to reconcile the conflicting results on the frequency of circulating MAIT cells in MS (Figure 2). The authors showed an IL18-driven activation and consequent CNS infiltration of CD8^+^ MAIT cells in MS, possibly causing reduced frequency in blood [74]. Another work corroborated the evidence of MAIT cells in MS brain infiltrates, by comparing lesional and peripheral blood TCR repertoires, finding massively expanded and longitudinally persisting T cell populations bearing canonical or atypical MAIT cell-related α chains in MS lesions [75].

Further implication of these unconventional T cells in MS pathophysiology came from a study by Willing et al., showing an IL7-associated, augmented type-17 differentiation of circulating MAIT cells in patients compared to controls [76]. Moreover, a recent study, demonstrating abnormal effectors and regulatory T cell subsets in pediatric-onset cases of MS, showed a prominent role of circulating pro-inflammatory CD8^+^CD161^high^ MAIT cells in children with MS compared to controls [77].

A longitudinal study proposed the follow-up of circulating MAIT cells as a biomarker of MS course (being their levels correlated to the dynamics of clinical and neuroimaging data), as well as a therapeutic target for the development of new treatment [78]. In this context, at least three works found changes in circulating MAIT cells before and after disease-modifying approaches (Figure 2). Non-myeloablative autologous hematopoietic stem cell transplantation in MS patients proved to deplete MAIT cells producing IL17 [79]. This finding was confirmed in a recent study using the common regimen with carmustine, etoposide, cytarabide, melphalan in therapy-refractory MS patients: the myeloablative BEAM approach led to the ablation of proinflammatory MAIT cells [80]. A reduction of CD8^+^CD161^+^ MAIT cells was also reported in MS patients after treatment with dimethyl fumarate, in a study aimed at investigating changes in immune cell subsets of MS patients, that were independent of the drug effects on the absolute lymphocyte count [81].

Recent works dealt with the possible relationship between MAIT cells and risk factors for MS development (Figure 2). Especially smoking, which is a recognized modifier of MS risk [82], seemed to affect the frequency of circulating MAIT cells. Specifically, a reduction of MAIT cell subsets was described in smokers in two distinct papers [83,84]. One of these reported on patients with primary progressive disease, and also in this context, the peripheral MAIT cells were clearly reduced in patients with respect to controls, irrespective of their smoking status and in apparent contrast to what was observed in most studies on a patient with relapsing-remitting MS [84]. Loss of circulating CD8^+^CD161^+^T cells, belonging to the MAIT cell subset, was confirmed in a recent work in patients with primary progressive MS [85]. Further investigations are necessary to clarify the dynamics of peripheral MAIT cells in MS, and the apparent alteration of this circulating subset in patients with progressive onset.

MAIT cells are involved in many inflammatory and autoimmune diseases other than MS [86]. Differences in MAIT cell frequency can be observed in the same condition, when different tissues are considered, mainly when peripheral blood and other disease-associated tissues are compared (Table 1).

As shown in the table, conflicting results regarding MAIT cell frequencies in different tissues can be observed in various diseases. This inconsistency may be due to the methodology used by the different research groups to identify MAIT cells: MR1 and the invariant TCRα chain expressed by MAIT cells are strikingly conserved among species, and are usually used as main features to identify this subset; however, differences in age, gender, obesity, smoking, and possible treatments, characterizing the populations analyzed, may play a role in the heterogeneity of some results [83,97]. In the present section, we summarized the most compelling evidence on the MAIT cell contribution to the development of SLE [87,88], RA [87,89], SS [90,91], T1D [92], and IBD [94], as examples of autoimmune multifactorial diseases involving a diffuse activation of the immune system and a gut microbiota dysbiosis to whom MAIT cells may be associated.

SLE and RA are autoimmune diseases sharing similar features involving chronic inflammation and activation of innate and adaptive immunity [98,99]. Recent studies showed an alteration of the gut microbiome [100,101,102] and frequency changes of MAIT cells associated with the disease course. MAIT cells defined as CD3^+^γσ^-^ T cells expressing TCR Va7.2 and CD161^high^ were analyzed in peripheral blood of SLE and RA patients, showing a significant reduction of percentage and absolute number when compared to healthy subjects. Moreover, the MAIT cell frequency significantly correlated with age and disease activity and was independent of drug treatments. Interestingly, in paired synovial fluids from RA patients, an increased number and percentage of MAIT cells compared to blood was observed [87]. In early untreated RA patients, the MAIT cells were mostly CD4^+^ and progressively moved to CD8^+^ with the disease progression, indicating a shift within the MAIT cell population [89]. In SLE blood samples an impairment of IFNγ production by MAIT cells was reported; the change is probably due to a defect in nuclear factor of activated T cell (NFAT)1 signaling [87]. Further investigations on SLE confirmed the reduction of frequency in blood, suggesting that this phenotype might be due to an increased level of cell death, rather than to a downregulation of surface markers [88].

A possible role of MAIT cells was recently studied in SS, one of the most prevalent rheumatic diseases [103,104]. Specifically, two papers showed a reduction of MAIT cells in peripheral blood and highlighted the presence of these cells in salivary glands from primary SS patients compared to healthy tissues [90,91]. Unlike other diseases, the MAIT cells in peripheral blood in SS patients showed a CD4^+^ phenotype and resulted phenotypically as well as functionally altered compared to healthy subjects [90].

T1D is an autoimmune disease where the pancreatic β-cells are destroyed by autoreactive immune cells [105]. Rouxel et al. suggest that MAIT cells may participate in this damage, by directly killing the pancreatic beta-cells in humans and in the NOD mouse model of T1D. This hypothesis is supported by data showing an increased activation status and migration of MAIT cells to inflamed tissues in the NOD mice [92]. Another group reported a decrease of CD8^+^ and double negative MAIT cells in peripheral blood of recent onset children compared to established T1D children [92], but no evidence was found for the presence of MAIT cells in the insulitic lesions of patients recently diagnosed with T1D [93].

In IBD, including CD and UC, the etiology is partly attributed to a dysregulated immune response to gut dysbiosis involving both innate and adaptive immune systems. Specifically, MAIT cells seemed to be involved in these processes, being activated and recruited towards the inflamed tissues [106]. However, other data showed a reduced frequency of MAIT cells in inflamed mucosae of patients with UC and CD with respect to non-IBD controls, probably due to an increased level of apoptosis in these cells [95]. The analyses performed on peripheral blood of IBD patients agreed on the reduction of MAIT cells with respect to non-IBD subjects [56,57,58], showing also a correlation between the drop of MAIT cells and the disease activity [96].

## 5. Conclusions

The overall evidence of the last decade favor the implication of MAIT cells and microbiota in several chronic inflammatory diseases, including MS, as discussed in the previous sections. The proinflammatory profile of MAIT cells, and in particular their frequent bias towards IL17 production, includes this T cell subset in a general peculiarity of the type-17 immune response, that not only plays a pivotal role in the protection against bacteria, fungi, and certain viruses but also contributes to dysfunctional responses in case of infection, autoimmunity, degenerative conditions, and cancer [107,108,109]. The search for molecular signatures to distinguish between beneficial and harmful MAIT cell subsets will be of invaluable value to identify candidate biomarkers and potential new therapeutic targets for MS and other inflammatory chronic diseases.

MAIT cells are considered critical for optimal mucosal responses to microbial infections, especially in the intestine environment. In autoimmune diseases, a crucial question remains the discovery of the mechanisms by which MAIT cells become activated and pathogenic even in apparent sterile conditions, such as in the brain. Far-from-gut effects of immune effectors activated by dysfunctional processes affecting bowel barrier, microbiota composition, and gut-brain axis were described in EAE [110,111,112], and are already accepted also for non-inflammatory neurological conditions [12,53]. These effects may also exert a protective role, as in the case of IgA-producing plasma cells that, mobilized from the gut, play a role in suppressing neuroinflammation [113]. In MS and other autoimmune conditions, further studies are needed to find correlations between intestinal permeability, dysbiosis, MAIT cell responses, and clinical-instrumental biomarkers in treated and treatment-naïve patients. They may help explain what activates MAIT cells in diseases not primarily infective, which subsets are potentially pathogenic, and their dynamics related to disease course and disease-modifying treatments.

## Figures and Tables

**Table 1:** MAIT cell frequency in autoimmune diseases in different tissues.

Diseases	MAIT Cells Frequency		References
	Peripheral Blood	Diseased Tissues	
SLE	↓	ND	[87,88]
RA	= ↓	↑ enriched in synovial fluids compared to PBMCs	[67,87,89]
SS	↓	↑ in labial salivary gland form primary SS patients compared to healthy tissues	[90,91]
T1D	↓ in recent-onset children compared to established T1D	ND	[92,93]
IBD	↓ in blood compared to healthy colons	↑ in inflamed colons compared to healthy colons ↓ in IBD inflamed colons with respect to colons from non-IBD	[94,95,96]
MS	↑↓ in RRMS ↓ in PPMS	↑ in CNS from MS patients compared to healthy tissues	[67,78]

SLE = Systemic lupus erythematosus; RA = Rheumatoid arthritis; SS = Sjögren syndrome; T1D = Type 1 diabetes; IBD = Inflammatory bowel disease; MS = multiple sclerosis; ↑ = increased frequency; ↓ = decreased frequency; = no change; ND = not detected; PBMCs = Peripheral blood mononuclear cells; RRMS = Relapsing-remitting multiple sclerosis; PPMS = Primary progressive multiple sclerosis; CNS = Central nervous system.

## Data Availability

Not applicable.
